# Alopecia in Patients with Autoimmune Hepatitis Treated by Tacrolimus Therapy

**DOI:** 10.5152/tjg.2024.24051

**Published:** 2025-01-01

**Authors:** Osman Yüksekyayla, Ersin Batıbay, Staffan Wahlin, Cumali Efe

**Affiliations:** 1 Department of Gastroenterology, Harran University, Şanlıurfa, Türkiye; 2 Division of Hepatology, Department of Upper GI Diseases, Karolinska Institutet and Karolinska University Hospital, Stockholm, Sweden

Dear Editor,

Tacrolimus has been successfully used in autoimmune hepatitis (AIH) as a second-line treatment option.^1-2^ Several side effects, such as nephrotoxicity, neurotoxicity, and metabolic effects have been reported. Tacrolimus-related alopecia is a rare side effect that has been reported in organ transplant recipients (kidney, liver, and pancreas).^3-4^ Only a few cases of tacrolimus-induced hair loss have been reported among tacrolimus-treated patients with AIH.^1^ We present 2 AIH patients who suffered from alopecia during tacrolimus therapy.

Case 1: A 26-year-old woman who had been diagnosed with AIH 5 years earlier was treated with a combination of corticosteroids, azathioprine (50-150 mg/day, 8 months), and 6-mercaptopurine (50 mg/day, 16 months) for 2 years, but her aminotransferase levels never reached normal levels. Tacrolimus 8 mg/day in addition to prednisolone 5 mg/day was used as a rescue therapy, which induced biochemical remission after 4 months of therapy. The serum tacrolimus levels were maintained at around 8 ng/mL. Ten months after the start of tacrolimus therapy, she presented with hair loss. Other potential causes of hair loss were excluded by evaluation of serum iron, vitamin B12, and vitamin D levels, and thyroid function tests. Alopecia areata was diagnosed by a dermatologist, and the patient’s hair loss was attributed to tacrolimus. All potential benefits and risks of tacrolimus therapy were explained to the patient, and it was decided to continue therapy, but with the dose of tacrolimus reduced to 4 mg/day. To prevent possible disease relapse, the dose of prednisolone was increased to 15 mg/day. Following this management, her hair loss reversed, and her aminotransferase levels remained within normal ranges. After 22 months of follow-up, she had a full head of hair, and her AIH was in complete biochemical remission on tacrolimus 4 mg/day and prednisolone 2.5 mg/day.

Case 2: A 52-year-old woman diagonsed with AIH had been treated with prednisolone and azathioprine (50-150 mg/day) for about 2 years. Due to multiple disease relapses despite good compliance to therapy, she was switched from azathioprine to tacrolimus. She maintained remission, and no relapse was observed during 8 months of therapy with tacrolimus 4 mg/day and prednisolone 5 mg/day. She reported experiencing hair loss. The patient was informed that her hair loss might be a side effect related to tacrolimus. Consequently, she discontinued tacrolimus therapy and switched to mycophenolate mofetil (MMF). She maintained AIH remission, and her hair loss completely reversed during 16 months of follow-up on MMF 1500 mg/day and prednisolone 2.5 mg/day.

Several studies have extensively evaluated extrahepatic manifestations in patients with AIH, but the exact number of patients affected by skin disorders was not reported.^5^ These results suggest that skin diseases are usually overlooked or underreported in patients with AIH. Serum zinc levels were not checked, which may be related to alopecia in our 2 cases. Importantly, both patients did not need zinc supplementation and were successfully managed by immunosuppressive modification.

It is not clearly known how tacrolimus induces alopecia, while topical application of tacrolimus is used to treat alopecia. Some studies have reported that tacrolimus is associated with the disruption of vascular endothelium, which diminishes blood flow to the hair follicles, consequently leading to alopecia.^3,6^ Tacrolimus-induced alopecia is managed by switching tacrolimus to cyclosporin or by reducing tacrolimus doses in the transplant setting. In AIH, tacrolimus is generally used for difficult-to-treat patients.^1,2^ Few alternative therapies are available when patients develop tacrolimus-related side effects. Our first case did not respond to standard AIH therapy, and obtaining remission was crucial because her laboratory and imaging features were suggestive of advanced liver disease. Therefore, the patient was convinced to maintain tacrolimus therapy, although with a reduced dose. This patient also desired pregnancy, and MMF was therefore not an option. The other patient was very concerned with the therapy-related cosmetic side effects but did agree to switch to MMF.

Cosmetic side effects are commonly observed in corticosteroid-treated patients with AIH. These side effects jeopardize patients’ compliance and represent challenges for clinicians in the management of AIH. The side effects of second-line therapies (MMF, tacrolimus or everolimus) have not been well documented in AIH. Therefore, we encourage hepatologists to report any suspected adverse effects induced by second-line therapies, regardless of their severity. Our 2 cases suggest that cosmetic side effects, such as alopecia, can occur due to tacrolimus therapy, and management should be carefully decided after weighing the benefits and potential side effects.

## Data Availability

All relevant data that support the findings are presented in the article.
